# Combined depletion of interleukin-1 and interleukin-6 does not exceed single depletion of interleukin -1 in TNF-mediated arthritis

**DOI:** 10.1186/ar3645

**Published:** 2012-02-09

**Authors:** Silvia Hayer, B Niederreiter, J Smolen, K Redlich

**Affiliations:** 1 Department of Internal Medicine III, Division of Rheumatology, Medical University of Vienna, Vienna, Austria

## Background

Previous studies demonstrated a regulatory role of interleukin 1 (IL-1) in inflammatory cartilage damage and bone destruction in human tumor necrosis factor transgenic (hTNFtg) mice, an animal model for Rheumatoid Arthritis (RA). Moreover, blocking of IL-6 has been shown to reduce local bone erosions in this model. Therefore we wanted to investigate the effect of a combined depletion of IL-1 and IL-6 on the development and severity of inflammatory, erosive arthritis.

## Methods

We first crossed IL1α and ß deficient (IL1-/-) mice with IL6-/- mice to generate IL1-/-IL6-/- double knockout mice. We next intercrossed these animals with arthritogenic hTNFtg mice to receive IL1-/-IL6-/-hTNFtg mice. We weekly assessed clinical signs of arthritis in hTNFtg, IL1-/-hTNFtg mice, IL6-/-hTNFtg mice and IL1-/-IL6-/-hTNFtg mice starting from week 4 after birth until week 16. We stained decalcified paw sections from all 4 genotypes with hematoxylin & eosin to determine the amount of inflammatory synovial pannus formation, with tartrate-resistant acid phosphatase (TRAP) to evaluate the number of synovial osteoclasts and the occurrence of subchondral bone erosions, with toluidine-blue to assess articular cartilage damage. Quantitative analysis of histopathological changes were performed using the Osteomeasure Software System.

## Results

We found a significant reduction in the clinical signs of arthritis, indicated by an increase of paw swelling and a decrease in grip strength, in IL1-/-IL6-/-hTNFtg mice when compared to their hTNFtg littermates. In line with these findings we observed a significant decrease in synovial inflammation in IL1-/-IL6-/-hTNFtg mice when compared to hTNFtg animals. Moreover, the number of synovial TRAP+ osteoclasts was markedly diminished in IL1-/-IL6-/-hTNFtg mice and reduced osteoclast formation, was accompanied by significantly less subchondral bone erosions. Additionally, we found a conserved articular cartilage structure showing almost no cartilage degradation in IL1-/-IL6-/-hTNFtg mice compared to their hTNFtg littermates. In IL1-/-IL6-/-hTNFtg mice clinical, as well as, histological signs of disease, including joint inflammation, bone destruction and cartilage damage were also significantly diminished when compared to IL6-/-hTNFtg mice. However, by comparing IL1-/-IL6-/-hTNFtg mice with IL1-/- hTNFtg mice we found a similar reduction on synovial inflammation, as well as subchondral bone erosions and articular cartilage destruction.

## Conclusion

The phenotype of IL1-/-IL6-/-hTNFtg mice does not differ from IL1-/-hTNFtg animals indicating no synergistic effects when IL-1 and IL-6 is simultaneously blocked in TNF-mediated arthritis.

